# Development of a bacterin–toxoid vaccine using a Korean isolate for protection against caseous lymphadenitis in goats

**DOI:** 10.1186/s13567-025-01685-8

**Published:** 2026-01-03

**Authors:** Hokeun Won, Eeuri Nam, Youngjung Shim, Ji-Youn Moon, Byeong-yeal Jung, Min-Kyoung Shin

**Affiliations:** 1 ChoongAng Vaccine Laboratories Co., Ltd., Daejeon, 34055 Republic of Korea; 2 Animal and Plant Quarantine Agency, Gimcheon, 39660 Republic of Korea; 3 Department of Microbiology and Convergence of Medical Science, College of Medicine, Gyeongsang National University, Jinju, 52727 Republic of Korea

**Keywords:** *Corynebacterium pseudotuberculosis*, caseous lymphadenitis, bacterin–toxoid vaccine, goat, sheep, field trial

## Abstract

Caseous lymphadenitis (CLA), a chronic infectious disease caused by *Corynebacterium pseudotuberculosis*, remains a major concern in small ruminant populations owing to its persistent nature and impact on animal productivity. Despite the availability of commercial vaccines, their efficacy in goats has been inconsistent and field-based evidence supporting their use remains limited. In this study, we report the development and evaluation of a novel bacterin–toxoid vaccine formulated with *C. pseudotuberculosis* strain 51-12A obtained from a Korean native goat. The toxoid, prepared from culture supernatant and enriched with immunoreactive proteins such as PLD, CP40, NanH, and PknG, was combined with formalin-inactivated bacterin to formulate a dual-component vaccine. Immunization trials in 3–4-month-old goats demonstrated that animals receiving the combined vaccine mounted strong anti-PLD responses (optical density, OD; OD ≥ 1.0), exhibited complete protection against experimental challenge with 1 × 10⁶ colony-forming units (CFU)/mL of homologous strain, and showed no evidence of internal abscesses. In addition, field studies conducted at three independent farms confirmed the safety and immunogenicity of the vaccine under practical conditions. Vaccinated animals maintained elevated antibody titers and remained clinically healthy, while unvaccinated controls developed CLA-associated abscesses. Comparable immune responses observed in sheep suggest potential cross-species applicability. These findings highlight the efficacy and field suitability of this goat-adapted vaccine and support its potential role in CLA control strategies for small ruminant production systems.

**Supplementary Information:**

The online version contains supplementary material available at 10.1186/s13567-025-01685-8.

## Introduction

Caseous lymphadenitis (CLA) is a chronic infectious disease affecting small ruminants, notably goats and sheep, across numerous regions [1]. The causative agent, *Corynebacterium pseudotuberculosis*, is capable of entering the host through minor skin breaches or mucosal contact, where it resists intracellular killing and remains within macrophages, and then via lymphatic circulation [2, 3]. This leads to chronic pyogranulomatous inflammation and the development of characteristic abscesses, most commonly in superficial lymph nodes and, in advanced cases, in visceral organs [3, 4]. In sheep, these lesions often display an onion-ring appearance due to concentric fibrous layers, whereas in goats, the lesions usually present as a dry, uniform purulent mass [5]. The disease results in major losses related to animal productivity, reproductive efficiency, and increased management costs [6, 7]. Although direct transmission to humans is infrequent, zoonotic potential has been documented, especially in individuals with occupational exposure [8–10].

Owing to the bacterium’s ability to survive within host cells, eradication through antimicrobial therapy alone is rarely successful. Another obstacle is that CLA abscesses are surrounded by thick fibrous capsules, which prevent antibiotics from reaching the bacteria effectively, even when laboratory tests show susceptibility [1]. Field trials have also demonstrated that, although some improvement can be achieved with drainage and antimicrobials, complete cure is uncommon and recurrence remains a problem [11]. Veterinary manuals further emphasize that long treatment courses are costly, impractical in farm conditions, and rarely prevent relapses [1]. Recently, an alternative approach using intra-abscess instillation of ozone or hydrogen peroxide was reported to show promising therapeutic effects in small ruminants [12], but this method remains experimental and has not yet been validated under field conditions. Taken together, these findings confirm that antibiotic therapy alone is not sufficient for reliable CLA control, underscoring the need for vaccination [1]. Most of these vaccines were originally developed for use in sheep and typically contain inactivated phospholipase D (PLD) from *C. pseudotuberculosis*, frequently combined with toxoids from *Clostridium* species [1, 13]. Field experience has shown that the protection offered by current CLA vaccines is incomplete [1]. In Australia, for example, Glanvac^®^ (Zoetis, Australia) has been in use for decades and is considered the main control tool; however, even under long-term vaccination programs, eradication has not been achieved [6, 14]. The vaccine reduces the frequency of lung lesions and slows the spread of infection at the flock level, but its protective efficacy in goats remains inconsistent [1]. In the USA, Caseous D-T^®^ (Colorado Serum Co., USA) combines *C. pseudotuberculosis* bacterin and toxoid with *Clostridium perfringens* and *Clostridium tetani* components [15]. It has been applied in sheep and goats but offers only partial protection and is often associated with injection-site abscesses and other side effects [1]. In Brazil, LinfoVac^®^ (Vencofarma, Brazil), based on a live attenuated strain originally isolated from a goat, is approved for both sheep and goats and showed partial protection under controlled conditions [1, 13]. Despite the availability of these products, none offer complete protection, and their performance in goats remains inconsistent [5]. Reports of adverse effects, such as injection-site abscesses, fever, and reduced milk yield, have raised concerns about vaccine safety, particularly in goat populations [5, 6]. Furthermore, most commercial vaccines are optimized for sheep and few have undergone rigorous field validation in goats.

Globally, CLA prevalence varies widely, ranging from 1.6% to over 45%, depending on the region: 8.9% of sheep and 1.6% of goats in Algeria, 2.5% of goats in Venezuela, 5.1% of goats in Thailand, 14.4% of goats in India, 37.9% of goats in Italy, 39.2% of goats in China, and 45% of sheep in the UK [16–18]. In Korea, CLA prevalence in goats has been estimated at 7.3%, but comprehensive epidemiological studies remain scarce [16, 19]. Most available data are limited to case reports or slaughterhouse surveys. Given that Korean native goats, particularly the black-coated variety, represent over 80% of the national goat population and are raised on more than 9000 farms, even moderate prevalence rates pose a considerable threat to the Korean goat industry [16, 20].

At present, South Korea lacks an officially registered vaccine against CLA. As a result, disease management relies on conventional measures such as culling and environmental sanitation. However, these approaches are often reactive and insufficient for preventing herd-level transmission, particularly among young animals that have lost maternal antibody protection. This highlights a clear need for the development of a vaccine adapted to local strains and goat-specific epidemiology.

**Figure 1:**
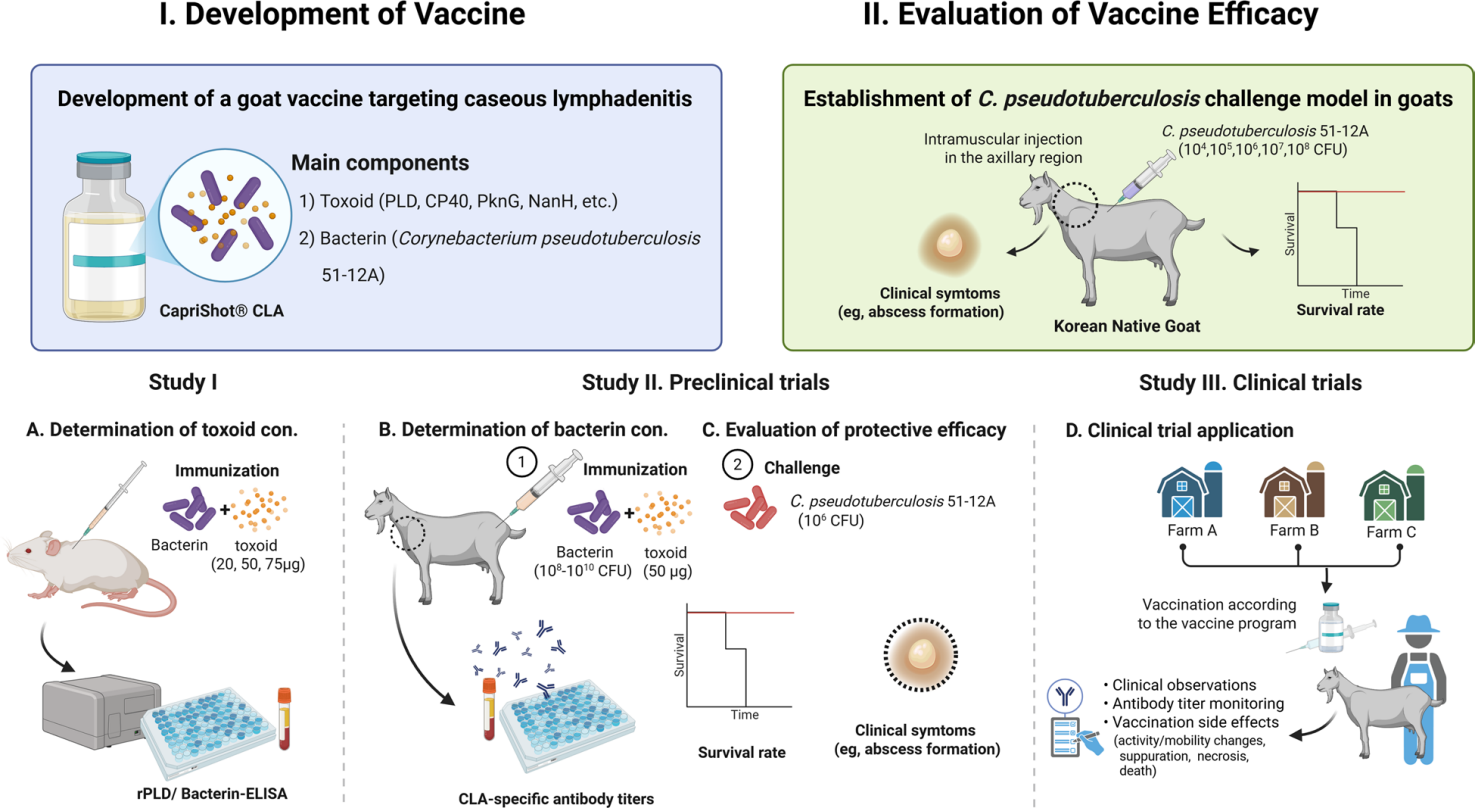
**Schematic diagram of the study to develop and evaluate a bacterin–toxoid vaccine against caprine caseous lymphadenitis (CLA) using a Korean isolate of *Corynebacterium pseudotuberculosis*.** The study was divided into two major phases: (i) vaccine development, including the production and optimization of bacterin and toxoid components derived from *C. pseudotuberculosis* strain 51-12A, and (ii) vaccine efficacy evaluation through experimental challenge and field application. Study **A**: the optimal toxoid dose was determined in BALB/c mice using a fixed bacterin concentration (1 × 10⁹ CFU) and various toxoid doses (20, 50, and 75 µg). Antibody responses to rPLD and whole cell bacterin were measured using enzyme-linked immunosorbent assay (ELISA). Study **B**: determination of optimal bacterin dose and protective efficacy in goats. Animals were immunized with various concentrations of bacterin (1 × 10⁸–10^1^⁰ CFU) and 50 µg of toxoid and then challenged with highly virulent *C. pseudotuberculosis* to evaluate immunogenicity and protective efficacy. Study **C**: field study involving two goat farms and one sheep farm to evaluate the safety and immunogenicity of the vaccine in a field environment.

This study aimed to develop and evaluate a CLA vaccine based on a *C. pseudotuberculosis* strain isolated from a Korean goat herd. In this study, we developed a vaccine containing toxoid and bacterin using the Korean goat isolate *C. pseudotuberculosis* 51-12A, and evaluated the composition of the vaccine and its immunogenicity and protective efficacy through laboratory challenge experiments and field trials. We established a *C. pseudotuberculosis* infection model in goats and further evaluated the vaccine safety and efficacy in an outdoor farm, thereby establishing a comprehensive CLA vaccine evaluation model platform (Figure 1). The goal is to provide a practical and evidence-based vaccination strategy to support CLA control efforts in Korea.

## Materials and methods

### Experimental animals, bacterial strain, and ethical approval

This study involved 56 healthy, CLA-seronegative Korean native goats aged 3–4 months and 80 specific-pathogen-free (SPF) BALB/c mice aged 6 weeks. The goats were sourced from local farms, and the mice were purchased from Samtako Bio Korea (Gyeonggi-do, South Korea). All animals were acclimatized for more than a week prior to experimentation to ensure suitability for the procedures. All animal procedures, including both murine and caprine experiments as well as the field trial, were reviewed and approved by the Institutional Animal Care and Use Committee (IACUC) of ChoongAng Vaccine Laboratory (approval no. 200211-01). In addition, the final clinical trial protocol was approved by the Animal and Plant Quarantine Agency of Korea (APQA) (approval no. Veterinary Pharmaceutical Management Division-5397, 24 April 2024). All experiments were conducted in accordance with relevant animal welfare regulations and ethical guidelines.

The bacterial strain used for antigen production and challenge experiments was *C. pseudotuberculosis* 51-12A, a clinical isolate derived from a Korean native goat with confirmed caseous lymphadenitis. The strain was provided by the Animal and Plant Quarantine Agency, Republic of Korea.

### Bacterial culture and toxoid preparation

*C. pseudotuberculosis* 51-12A was cultured in Brain Heart Infusion broth supplemented with 0.05% Tween 80 (BHI-T) at 37 °C under shaking conditions (180 revolutions per minute [rpm]) for 48 h. The culture was filtered through a 0.2-µm membrane to separate bacterial cells and culture supernatant. Each fraction was inactivated by treatment with 0.2% formaldehyde at 37 °C for 96 h. After inactivation, the bacterial cells and supernatant were washed repeatedly with sterile PBS to remove any residual formaldehyde, and the residual formalin content in the final preparations was confirmed to be below 0.2% according to the national testing standard. The inactivated bacterial cells (bacterin) and toxoid derived from the culture supernatant were used individually or in combination for vaccine formulation.

The protein concentration of the toxoid was determined using the Pierce™ Coomassie Plus (Bradford) Assay Reagent (Thermo Fisher Scientific, Grand Island, NY, USA) according to the manufacturer’s instructions, with bovine serum albumin as the standard and absorbance measured at 595 nm. To assess the antigenic profile of the toxoid, protein samples from the culture supernatant were separated by 12% sodium dodecyl–sulfate polyacrylamide gel electrophoresis (SDS–PAGE) and transferred to a nitrocellulose membrane. Membranes were blocked overnight at 4 °C using 5% skim milk in phosphate-buffered saline (PBS), followed by three washes with PBS containing 0.1% Tween 20 (PBS-T). Primary antibody incubation was performed with serum from immunized mice (1:50 dilution in 0.5% skim milk) for 2 h at room temperature at 50 rpm on a rotary shaker. After washing, membranes were incubated for 1 h with alkaline phosphatase (AP)-conjugated rabbit anti-goat IgG (1:1000 dilution, Sigma, St. Louis, MO, USA) in 0.5% skim milk. Antigen–antibody reactions were visualized using a nitro blue tetrazolium/5-bromo-4-chloro-3-indolyl phosphate (NBT/BCIP) substrate kit (Bio-Rad, Hercules, CA, USA).

### Mouse immunogenicity test for antigen dose optimization

To optimize toxoid antigen dosage, a total of 80 healthy SPF BALB/c mice (15–20 g) were randomly assigned to eight groups (*n* = 10 per group). Mice received two subcutaneous injections of 0.1 mL each at a 2-week interval. Serum samples were collected 2 weeks after the second immunization for antibody titers analysis. The experimental design included vaccine formulations containing 1 × 10⁹ CFU inactivated bacterial cells combined with varying doses of toxoid (25, 50, or 75 µg), as well as bacterin-only, toxoid-only, and unvaccinated control groups.

Serum antibody titers were measured using a commercially available ELISA kit (ELITEST CLA, Hyphen Biomed, Neuville sur Oise, France), which utilizes recombinant PLD (rPLD) protein as the coating antigen, and a custom whole-cell ELISA using formalin-inactivated *C. pseudotuberculosis* bacterin. Since the commercial kit was originally designed for goats, the protocol was modified for murine sera. On the basis of optimization, the following conditions were established: (i) the mouse serum dilution was fixed at 1:100; (ii) the conjugate concentration was set at 1:10,000 using goat anti-mouse IgG (H + L) horseradish peroxidase (HRP) conjugated antibody (ThermoFisher Scientific); and (iii) clear separation was observed between vaccinated mice (optical density, OD; OD consistently > 0.5) and unvaccinated controls (mean OD 0.11). These results confirmed the reliability of using OD comparisons for immunogenicity screening in mice.

The custom whole-cell ELISA was developed by coating 96-well microplates with disrupted formalin-inactivated *C. pseudotuberculosis* cells. Bacterial cells (1 × 10^1^⁰ CFU/mL) were suspended in carbonate-bicarbonate buffer and mechanically disrupted using pulsed sonication (50% power, 2 s on/5 s off, 5–8 min total). Each well was coated with 100 µL antigen suspension (approximately 10 µg/mL) and incubated overnight at 25 °C. After washing with 0.1% PBS-T, wells were blocked with 5% skim milk at 37 °C for 2 h. Mouse serum samples were serially diluted to a final 1:100 dilution using 3% skim milk and applied in duplicate to antigen-coated wells. Following a 1-h incubation at 37 °C, the wells were washed and incubated with HRP-conjugated goat anti-mouse IgG (1:10000 dilution in 3% skim milk) for 30 min at 37 °C. After the final wash step, 3,3′,5,5′-tetramethylbenzidine (TMB) substrate was added (100 µL/well, Surmodics IVD, Inc., Eden Prairie, MN, USA) and allowed to react for 10 min at room temperature. The reaction was terminated with 100 µL of stop solution (2 M H_2_SO_4_), and absorbance was measured at 450 nm within 10 min.

### Goat challenge model establishment

To establish a reliable challenge infection model, 24 healthy, CLA-negative Korean native goats aged 3–4 months were divided into six groups (*n* = 4 per group). Each group received an intramuscular injection of 1.0 mL of *C. pseudotuberculosis* 51-12A at graduated concentrations of 1 × 10^4^, 1 × 10^5^, 1 × 10⁶, 1 × 10⁷, or 1 × 10⁸ CFU/mL, while one group served as an uninfected control. The injections were administered intramuscularly into the axillary region using a 20-gauge needle to ensure uniform delivery across animals. Animals were observed for 4 weeks post inoculation for clinical signs and mortality. Surviving goats were necropsied to assess internal abscess formation in organs such as liver, lungs, kidneys, and lymph nodes. On the basis of these observations, the challenge dose for efficacy testing was set at 1 × 10^6^ CFU/mL.

### Goat immunization and efficacy evaluation

A total of 32 CLA-negative Korean native goats aged 3–4 months were divided into eight groups (*n* = 4 per group). G1, G3, and G5 received only bacterin at doses of 1 × 10^8^, 1 × 10^9^, and 1 × 10^10^ CFU, respectively. G2, G4, and G6 were administered the same bacterin doses as G1, G3, and G5 but with an additional 50 µg of toxoid. G7 served as the challenge control (nonimmunized but challenged), and G8 served as the nontreated control (nonimmunized and unchallenged). The goats were vaccinated intramuscularly in the axillary region with 1.0 mL of each vaccine formulation, all containing 20% aluminum hydroxide gel as an adjuvant. Booster immunizations were administered 4 weeks later, followed by a challenge with 1 × 10^6^ CFU/mL *C. pseudotuberculosis* 51-12A at the same site at 3 weeks post boost. Serum samples were collected weekly to monitor antibody responses using a commercial ELISA kit (ELITEST CLA, Hyphen BioMed), and results were calculated according to the manufacturer’s instructions. Animals were monitored for clinical signs and mortality for 4 weeks post challenge. At the end of the study period, surviving goats were necropsied to evaluate internal abscess formation. The vaccine candidate used in this study was developed by ChoongAng Vaccine Laboratory Co., Ltd. (Daejeon, South Korea) as a potential commercial product. Its immunogenicity and protective efficacy were independently evaluated under both laboratory and field conditions.

### Field trial and clinical observation

This study was conducted to evaluate the immunogenicity and safety of the vaccine confirmed in laboratory conditions in an actual farm environment. A total of three farms, farm A (200 goats), farm B (200 goats), and farm C (200 sheep), were selected as the subjects of the study. A total of 15 animals (ten vaccinated and five nonvaccinated control groups) were tested for the safety and immunogenicity of the vaccine. All animals used in the study were healthy individuals aged 3 months or older, and the vaccine was administered intramuscularly at 1.0 mL per animal for the first time, followed by a booster vaccination 4 weeks later. This field study consisted of a vaccine safety confirmation test and an immunogenicity confirmation test. For the vaccine safety confirmation test, each animal was observed for reactions at the injection site and systemic clinical symptoms for 21 days after vaccination to evaluate the occurrence of vaccine-related adverse reactions. For the vaccine immunogenicity test in outdoor subjects, antibody responses were evaluated by measuring anti-PLD IgG antibody titers using a commercial ELISA kit (ELITEST CLA, Hyphen Biomed). Antibody titers were measured until 11 or 15 weeks after vaccination, depending on the schedule of each farm.

All animals used in the test were previously bred on each farm. To minimize stress owing to environmental changes, the breeding location was not changed, and the experimental conditions were standardized by maintaining the same amount of feed used previously. This test was conducted as part of a preclinical trial to evaluate the practical applicability and effectiveness in domestic fields, and all procedures were strictly documented and managed according to the approved test plan. All procedures were conducted by a certified investigator under the supervision of an authorized clinical trial institution designated by APQA.

### Statistical analysis

For the laboratory challenge experiments (*n* = 4 per group), data are displayed as individual values with the median. Given the small sample size (*n* = 4 per group) and the limited reliability of normality testing, nonparametric analyses were applied. Pairwise comparisons were conducted using the Mann–Whitney *U* test, and multiple group comparisons were performed using the Kruskal–Wallis test followed by Dunn’s post hoc procedure. Exact *p*-values, effect sizes (mean rank differences and *U* values), and false discovery rate (FDR)–adjusted *q*-values are reported. For the field trial experiments (*n* = 10 per group), data are presented as mean ± standard deviation (SD). Group comparisons were performed using Student’s *t*-test, and a *p* < 0.05 was considered statistically significant. Statistical analyses were conducted using GraphPad Prism software (version 9.5.0, GraphPad Software, USA).

## Results

### Production of toxoid secreted by *C. pseudotuberculosis*

**Figure 2:**
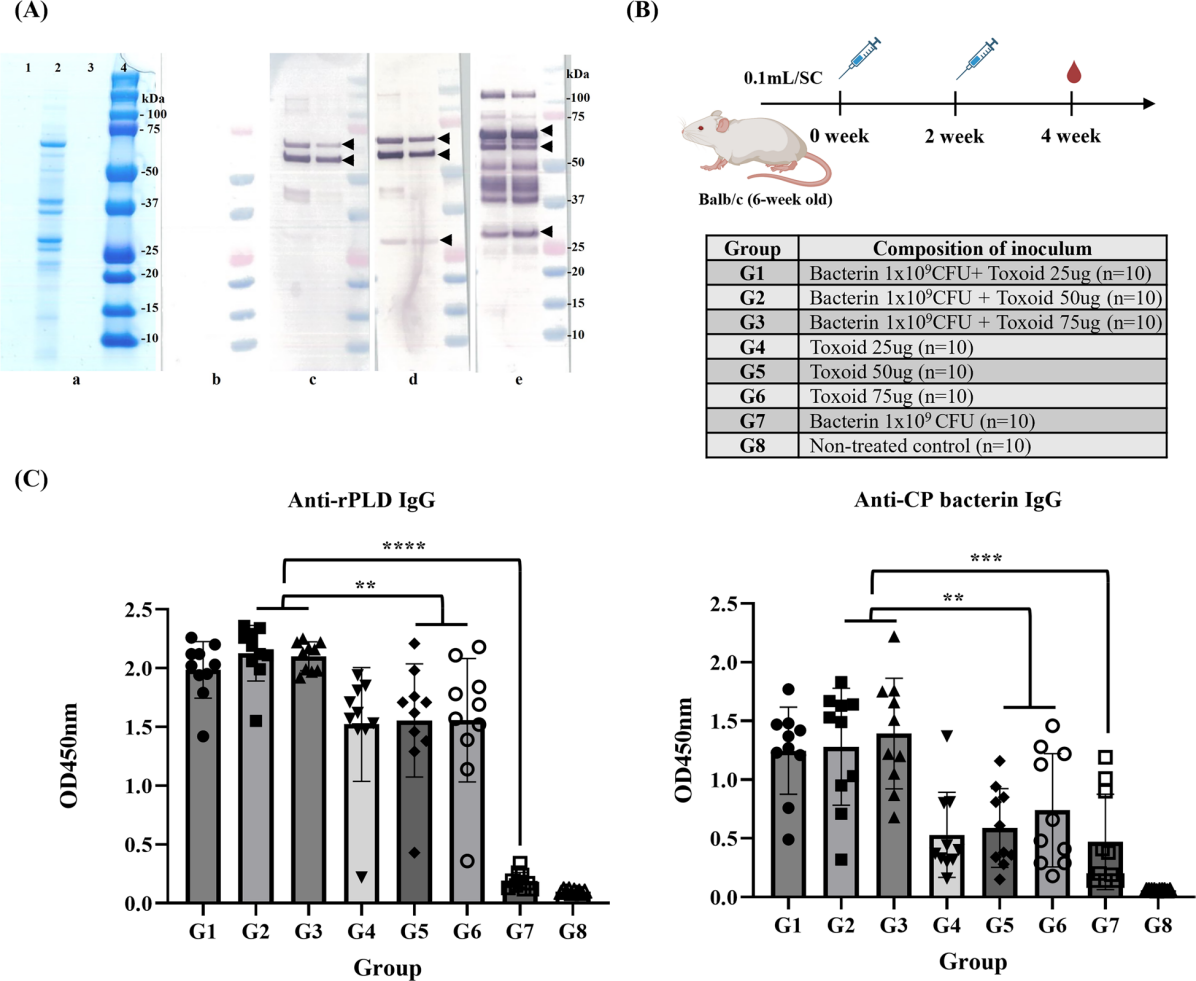
**Production of *Corynebacterium pseudotuberculosis*** **toxoid antigen and determination of optimal toxoid antigen concentration. A** SDS–PAGE and Western blot analysis of proteins secreted by *C. pseudotuberculosis* strain 51-12A. **a** SDS–PAGE: lane 1, BHI-T medium before incubation; lane 2, supernatant after incubation; lane 3, PBS-T buffer; lane 4, molecular weight marker. Distinct bands at ~32 kDa (PLD) and ~40 kDa (CP40) were detected only in the supernatant after incubation. **b**–**e** Western blot using sera from immunized mice with: **b** PBS (negative control); **c** bacterin alone; **d** bacterin + recombinant PLD (rPLD); **e** bacterin + toxoid. **B** Immunization schedule for optimizing toxoid dose in mice. BALB/c mice were vaccinated subcutaneously at 2-week intervals with a mixture of bacterin (1 × 10⁹ CFU) and 20, 50, or 75 µg of toxoid. **C** Serum antibody titers were measured 2 weeks after the second vaccination using a commercial rPLD-based ELISA kit (ELITEST CLA, Hyphen Biomed) and an in-house whole-cell ELISA. A significant increase in antibody titers was observed in the toxoid combination group. ^*^p < 0.05; ^**^p < 0.01; ^***^p < 0.001; ^****^p < 0.0001.

For toxoid production, the culture supernatant of *C. pseudotuberculosis* 51-12A, a representative Korean native goat isolate from domestic field cases, was analyzed by SDS–PAGE and Western blot. As shown in Figure 2A-a, a distinct protein band was observed only in the culture supernatant, and no detectable band was observed in the preculture medium or PBS-T control.

Western blot analysis was performed using sera obtained from mice immunized with various vaccine formulations, including bacterin alone, bacterin + rPLD, bacterin + toxoid, and a negative control. The sera from mice immunized with bacterin alone showed a specific reaction to a protein band between 50 and 75 kDa (Figure 2A–C). The group immunized with bacterin + rPLD showed immunoreactivity not only to the bacterin-related band but also to a distinct band corresponding to PLD at 32 kDa (Figure 2A–D). Mice receiving the bacterin + toxoid formulation showed reactivity to multiple bands, including the band corresponding to PLD (32 kDa) and additional bands representing other exotoxins (Figure 2A–E). This culture supernatant contained proteins secreted during bacterial growth, including several immunogenic proteins, including the major immunogenic protein, PLD.

### Determination of the optimal toxoid antigen dose in a murine model

A dose optimization study was performed in mice to evaluate the contribution of the toxoid components to immunogenicity. The experimental vaccine formulations contained a fixed concentration of bacterin (1 × 10^9^ CFU) and toxoid containing various doses of secreted proteins (25, 50, or 75 µg). Additional groups were administered toxoid alone or bacterin alone, and an unvaccinated group served as a negative control (Figure 2B). Serum antibody responses were assessed using two ELISA platforms: a commercially available rPLD-ELISA kit (ELITEST CLA) and a custom whole-cell ELISA coated with formalin-inactivated *C. pseudotuberculosis* bacterin. Mice immunized with bacterin + toxoid (G1–G3) showed significantly higher antibody titers in both ELISAs compared with all other groups (*p* < 0.01) (Figure 2C). Although no statistically significant differences were observed among the three toxoid doses in rPLD-ELISA (*p* = 0.983) or CP-ELISA (*p* = 0.486), a trend toward higher antibody titers was observed in groups receiving 50 µg or more, indicating enhanced immunogenicity at higher antigen concentrations. Although the toxoid-only groups (G4–G6) also showed seroconversion, the mean OD values ​​were consistently lower than those observed in the corresponding bacterin + toxoid groups. Pairwise comparisons between matched groups (i.e., G1 versus G4, G2 versus G5, G3 versus G6) revealed significantly higher responses (*p* < 0.05) in the combination formulation, confirming the synergistic immunostimulatory effect of bacterin (Figure 2C). Interestingly, the bacterin-only group (G7) had approximately 30% of the cells with OD ≥ 0.5 in the whole-cell ELISA, but no detectable reactivity in the rPLD-based assay, confirming the low immunogenicity of bacterin-only administration (Figure 2C).

### Establishment of a *C. pseudotuberculosis* challenge model in goats

To develop a robust challenge model in goats for vaccine efficacy evaluation, a dose-titration study was performed using *C. pseudotuberculosis* strain 51-12A obtained as a field isolate from the APQA of Korea. A total of 24 healthy Korean native goats, aged 3–4 months and confirmed as CLA seronegative by ELISA, were randomly assigned to six groups. Each goat was inoculated into the axillary muscle with 1.0 mL of the strain at a concentration of 1 × 10^4^–1 × 10^8^ CFU/mL. This route was chosen to create a model that closely mimics the natural pathogenesis of CLA, which typically invades through a skin wound and causes infection in the local lymph nodes. The animals were observed weekly for 4 weeks to monitor clinical signs and mortality. Typical clinical signs of CLA, including enlargement and abscess formation in superficial lymph nodes, as well as systemic manifestations such as changes in body condition, respiratory distress, and depression, were specifically checked during the observation period. After the observation period, all animals were necropsied to check for abscesses in internal organs and lymphoid tissues. Following experimental challenge, mortality patterns varied depending on the inoculum dose (Additional file 1). All goats challenged with 1 × 10^8^ CFU/mL (G1) and 1 × 10^7^ CFU/mL (G2) died by week 2, and no significant abscesses were detected at necropsy. In contrast, three of four goats in the 1 × 10^6^ CFU/mL group (G3) died during weeks 2–3, while the single surviving goat consistently exhibited internal abscess formation. At lower doses, single deaths were recorded in the 1 × 10^5^ CFU/mL (G4) and 1 × 10^4^ CFU/mL (G5) groups, occurring at weeks 2 and 4, respectively. Abscesses were consistently observed in survivors (Additional file 1). Lesions were mainly located in the lungs and peripheral lymph nodes, especially in the mandibular and parotid lymph nodes. This pattern was confirmed in all other groups. Among the survivors who were autopsied, abscess formation was most frequently observed in lung tissue and cranial lymph nodes (Additional file 1). On the basis of the results of the characteristics of the challenge dose, mortality, and lesion occurrence, a dose of 1 × 10^6^ CFU/mL was selected as the standard challenge dose for subsequent inoculations. In addition, abscess formation in the lung and mandibular or parotid lymph nodes were assumed as clinical symptom variables to evaluate pathogenicity and vaccine-induced protection.

### Evaluation of vaccine-induced immunity and protective efficacy in goats

**Figure 3:**
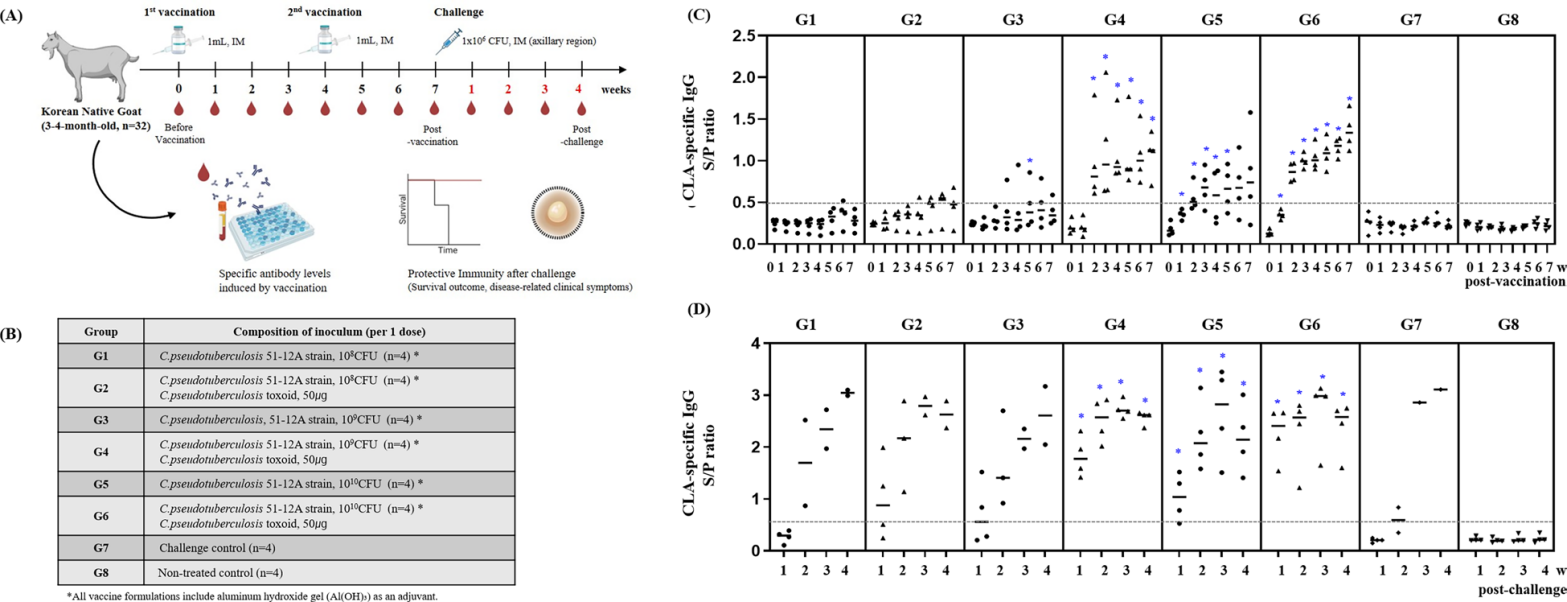
**Immunogenicity and antibody kinetics of candidate vaccines in goats. A** Experimental scheme of goat immunization and challenge. Korean native goats (3–4 months old) were immunized intramuscularly with bacterin at different doses (1 × 10⁸–10^1^⁰ CFU) with or without 50 µg of toxoid. **B** Antibody titers were monitored weekly from baseline (week 0) to 7 weeks post vaccination using an rPLD-based ELISA kit (ELITEST CLA). **C** Anti-PLD antibody titers were measured again 1–4 weeks after challenge. Each symbol represents an individual goat, and horizontal lines indicate the median per group. Statistical analysis was performed using the Mann–Whitney *U* test (for two-group comparisons) or the Kruskal–Wallis test followed by Dunn’s post hoc test (for multiple groups).

**Figure 4:**
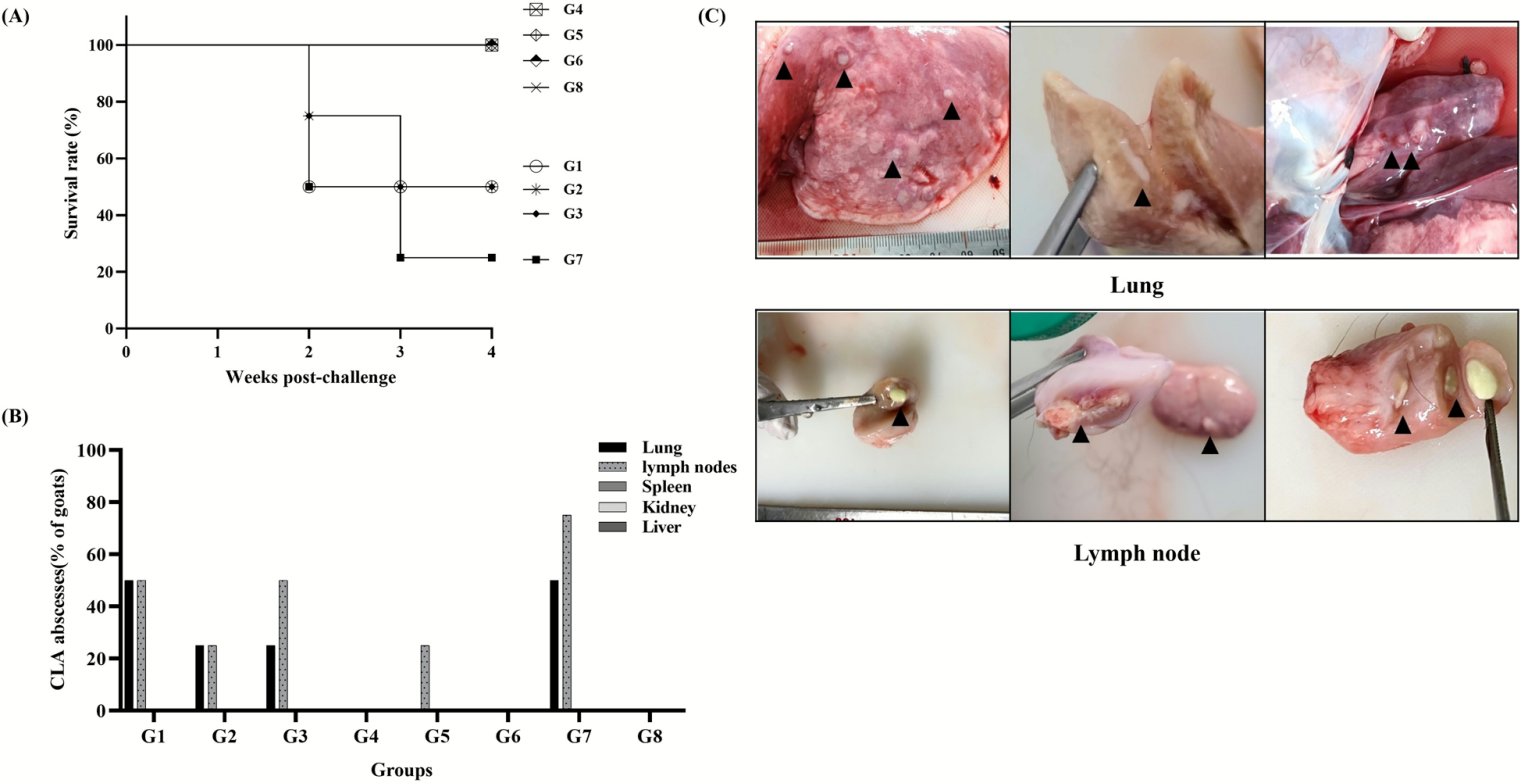
**Protective efficacy of vaccine candidates in a goat challenge model. A** Survival rates by vaccine group (G1–G8) during the 4-week observation period after challenge. **B** Incidence of CLA-related abscess formation in lung, lymph node, spleen, kidney, and liver. **C** Representative macroscopic lesions: lung abscess (upper panel) and lymph node abscess (lower panel) showed typical caseous morphology.

To evaluate the immunogenicity and protective efficacy of the candidate formulations, 32 healthy Korean native goats (3–4 months old) confirmed to be CLA antibody-negative were divided into eight groups (*n* = 4 per group). The animals were intramuscularly inoculated with 1.0 mL of the assigned vaccine, and a booster vaccination was performed 4 weeks later. A total of 3 weeks after the second vaccination, all goats were challenged intramuscularly with *C. pseudotuberculosis* 51-12A (1 × 10^6^ CFU/mL) in the axillary area. Anti-PLD ELISA kit (ELITEST CLA) was used to monitor antibody titers weekly before and after the challenge, and the clinical signs and survival rates of the animals were observed for 4 weeks (Figure 3A). Groups G4 and G6, which were administered a combination of 1 × 10^9^ or 1 × 10^10^ CFU of bacterin and 50 µg of toxoid, showed complete seroconversion at 2 weeks after vaccination. Mann–Whitney *U* tests confirmed that antibody titers in these groups were significantly higher than in controls from 2WPI onward (e.g., G4: *U* = 0.000, *p* = 0.0286, *q* = 0.0346; G6: *U* = 0.000, *p* = 0.0286, *q* = 0.0385). In contrast, goats administered bacterin-only vaccines (G1 and G3) showed delayed and partial seroconversion, which did not reach statistical significance compared with controls (*p* > 0.05). Specifically, in G3 (1 × 10^9^ CFU of bacterin), one goat showed seroconversion with an OD of 0.5 or higher after the first vaccination and three goats after the booster, whereas no consistent change was observed in G1 (1 × 10^8^ CFU of bacterin). Group G2, which were administered the same dose of bacterin as G1 with added toxoid, showed slightly improved seroconversion; however, the average antibody level was low, approximately OD 0.5 or lower (Figure 3C). After challenge vaccination, the G4 and G6 vaccine groups showed high protective immunity from the first week and showed high antibody titers exceeding OD2.0 (Figure 3D). Statistical analysis again confirmed significant differences compared with controls (*p* = 0.0286, *U* = 0.000), whereas bacterin-only groups (G1–G3) and low-dose combinations did not show significant protection. A high mortality rate of 50–75% occurred in the unvaccinated control group (G7) and the vaccine groups G1–G3 with no toxoid or low bacterin concentrations (Figure 4A).

However, no mortality or internal abscess was observed in G4 and G6 with toxoid and bacterin concentrations of 50 µg or more. There were no deaths in the G5 group, which received only 1 × 10^10^ CFU of bacterin, but lymph node abscesses were identified at necropsy (Figure 4B). The lesions were primarily found in the lungs and cranial lymph nodes, especially the mandibular and parotid lymph nodes. In the case of abscesses, round, cheese-shaped abscesses were formed on the surface or inside of the lymph nodes, and in the case of the lungs, abscesses were formed on the surface and inside of the cusp, heart, and diaphragmatic lobes (Figure 4C).

Taken together, these results demonstrate that the addition of toxoid was essential for generating robust immune responses. The G4 formulation (1 × 10^9^ CFU bacterin + 50 µg toxoid) consistently achieved 100% seroconversion, complete survival, and the absence of CLA lesions, supported by statistically significant differences compared with controls (Mann–Whitney *U* = 0.000, *p* = 0.0286, *q* = 0.0346). Considering both immunogenicity and protection data, the formulation (G4) containing 50 µg toxoid and 1 × 10^9^ CFU of bacterin was determined to be the optimal vaccine condition. This group consistently demonstrated 100% seroconversion, complete survival, and the absence of any CLA-related lesions after following challenge.

### Field application test and clinical observation

**Figure 5:**
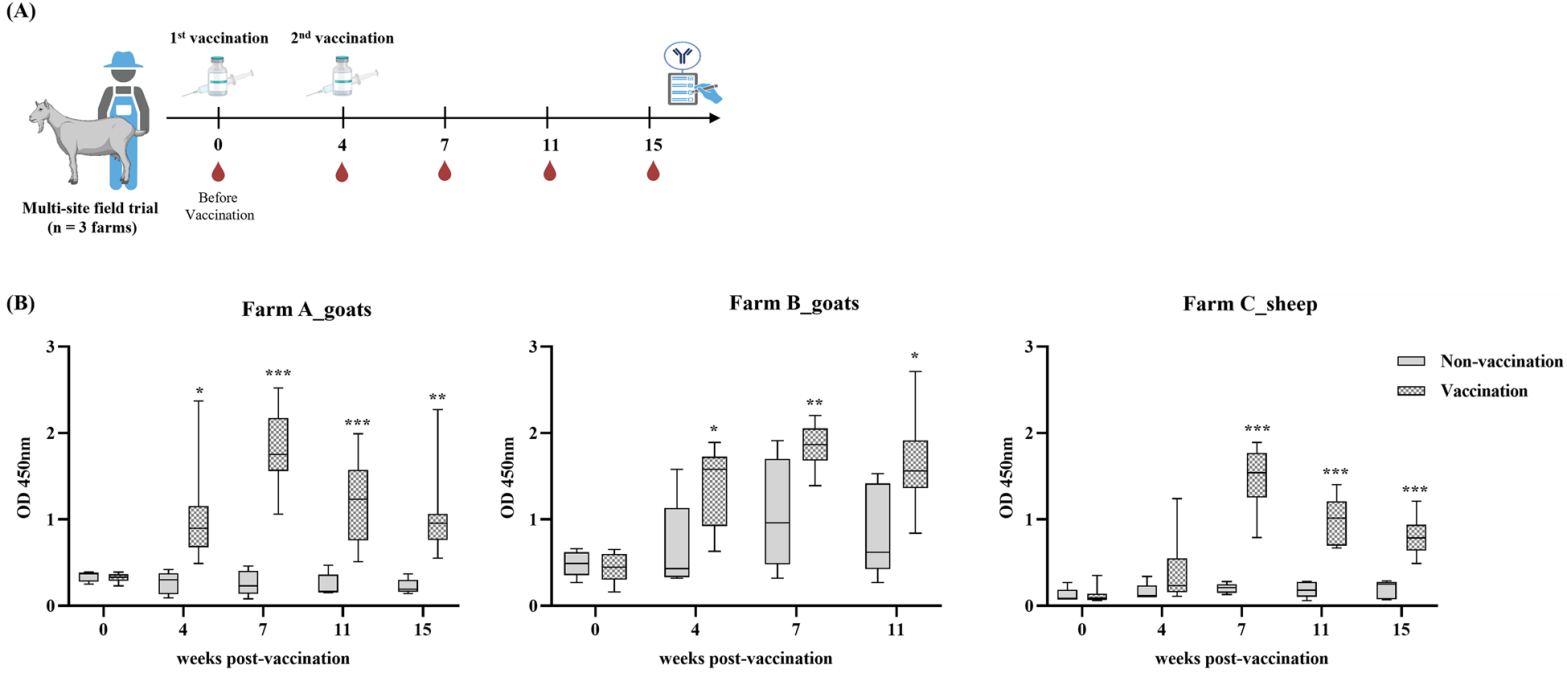
**Field trial evaluation of vaccine safety and immunogenicity. A** Field vaccination and sampling schedules on three farms (two goats, one sheep). Each animal received a primary and booster vaccination of the test vaccine at 4-week intervals. **B** Anti-PLD antibody titers were measured from week 0 to week 15 after vaccination using a commercially available rPLD-based ELISA kit (ELITEST CLA). Antibody titers in vaccinated animals increased significantly on all farms. ^*^p < 0.05; ^**^p < 0.01; ^***^p < 0.001; ^****^p < 0.0001.

This test was conducted on three farms to evaluate the safety and immunogenicity of the test vaccine in an actual breeding environment. On each farm, a total of 15 animals, consisting of 10 vaccinated and 5 nonvaccinated as control animals, were injected intramuscularly with the vaccine, and a booster vaccination was performed 4 weeks later (Figure 5A).

From the first vaccination until 3 weeks after the booster, no adverse reactions, such as mobility, activity, suppuration, necrosis, or death were observed on any of the farms (Additional file 2). However, on farm B, lesions indicative of caseous lymphadenitis were confirmed in two out of five goats in the nonvaccinated control group. One animal developed abscesses on both sides of the face 31 days after the first vaccination, and the other developed an abscess on the buttocks 45 days after the first vaccination. No similar lesions were observed in the vaccinated group (Additional file 2). Comparison of body weights before and 7 days after vaccination showed similar levels of weight gain in both the vaccinated and control groups, with no significant differences (data not shown). As a result of antibody titer analysis using ELISA, a clear increase in antibody titer and 100% seroconversion were confirmed in all farms. On farm A, all goats converted to seropositive 4 weeks after the first vaccination, and the antibody titer peaked 3 weeks after the second vaccination (week 7) and was maintained until week 11. From week 7–15, significantly higher antibody titers were observed in vaccination groups, compared with controls (*p* < 0.05) (Figure 5B). On farm B, some goats in the control group were antibody positive probably owing to natural infection, but all animals in the vaccinated group showed seroconversion at the fourth week post vaccination, reached the peak antibody titer in week 7, and was maintained until week 11. Even though natural infection occurred, the difference in antibody titers between the control group and the vaccinated group showed a statistically significant difference (*p* < 0.05) (Figure 5B). In the nonvaccinated control group, goats with higher antibody titers also presented CLA-compatible abscesses identified by clinical examination, indicating that seropositivity was accompanied by clinical signs of infection. On farm C (sheep), antibody titers gradually increased after the first vaccination, and 100% converted to positive 3 weeks after the second vaccination (week 7), and the antibody titers were maintained stably until week 11. The difference in antibody titers compared with the control group was statistically significant (*p* < 0.001) (Figure 5B).

## Discussion

Despite decades of research into CLA control, most vaccine development has centered around ovine use, with considerably less data available for goats [5]. Commercial vaccines such as Glanvac^®^ (Australia), Caseous D-T^®^ (USA), and LinfoVac^®^ (Brazil) are generally formulated using PLD-based antigens, often in combination with clostridial toxoids [1, 13]. However, these products were largely optimized for sheep, and their efficacy and safety profiles in goats remain inadequately characterized. Field reports describe inconsistent immune responses and adverse effects, including localized abscesses and reduced productivity. Moreover, few have been thoroughly evaluated under practical farming conditions for caprine application. Recent advances in vaccine research have explored a range of platforms beyond traditional bacterins and toxoids. Subunit formulations expressing recombinant antigens such as rPLD, CP40, or rCP09720, DNA-based vaccines targeting *pld* or other virulence genes, and vectored systems utilizing *Mycobacterium bovis* Bacillus Calmette–Guérin (BCG) as a delivery platform have all been investigated [1, 21, 22]. While these approaches show promise in murine models, very few have progressed to robust trials in target livestock species [1]. Most notably, there remains a critical gap in large-scale studies that assess both immunogenicity and safety in goats under field conditions. In this study, we developed a vaccine using a crude toxoid derived from culture supernatant and inactivated bacterin, both sourced from a *C. pseudotuberculosis* 51-12A strain recovered from a Korean native goat. The developed vaccine demonstrated both safety and significant protective effects in immunogenicity experiments in a domestic goat infection model, as well as in actual farm field tests, which is significant in that it complements the limitations of the existing CLA vaccine and presents a practical alternative as a preventive strategy optimized for goats.

*C. pseudotuberculosis* 51-12A used in this study belongs to the biovar ovis and possesses all 18 genes commonly associated with virulence, including *pld*, *fag* operon components, sigma factors E (*sigE*), tip protein C (*spaC*), zinc-dependent superoxide dismutase (*sodC*), protein kinase G (*pknG*), neuraminidase (*nanH*), oligopeptide permease A (*oppA*), and others (data not shown). The toxoid produced using this strain showed the most prominent bands among proteins by size, which were PLD of 32 kDa and CP40 of 40 kDa, and protein bands were identified between 50 and 70 kDa and were estimated to be immune-response proteins of various sizes, such as PLD (32 kDa), CP40 (40 kDa), NanH (72 kDa), PknG (83 kDa), SpaC (86 kDa), and SodC (18 kDa) (Figure 2A). Vaccine strategies relying solely on PLD or attenuated strains have been reported to offer partial protection but may be insufficient in completely controlling visceral or nodal lesions under certain conditions [5, 23]. In this study, when culture supernatant-derived toxoid (including PLD and many secreted proteins) and bacterin providing cellular antigens were co-administered, goats in G4 and G6 developed significantly stronger antibody responses compared with controls from 2 weeks post vaccination onward (e.g., G4: Mann–Whitney *U* = 0.000, *p* = 0.0286, *q* = 0.0346) (Figure 3C). These groups also achieved 100% survival and showed no CLA-compatible abscesses following challenge (Figure 4). The successful protection conferred by our dual-component vaccine can be attributed to complementary humoral and cellular immune pathways. The culture supernatant-derived toxoid, enriched with exotoxins such as PLD and CP40, is designed to induce neutralizing antibodies against secreted virulence factors. Previous studies have shown that toxoid PLD stimulates strong humoral responses and enhances nonspecific innate immunity when combined with bacterin component [24, 25]. In contrast, the bacterin component, composed of inactivated bacterial cells, primarily promotes robust Th1-type cellular immunity—characterized by IFN-γ and nitric oxide production—that is essential for controlling facultative intracellular pathogens such as *C. pseudotuberculosis* [26].

Indeed, our mouse model demonstrated a synergistic response: groups receiving both bacterin and toxoid (G1–G3) showed significantly higher antibody titers than either component alone, suggesting enhanced humoral activation (Figure 2C). In caprine trials, the G4 (bacterin + toxoid) formulation elicited particularly strong anti-PLD immune responses (S/P ratio ≥ 0.5), with no abscesses and no mortalities (Figures 3C, 4). These results align well with previous data indicating that vaccines combining toxoid and bacterin not only enhance specific antibody production but also activate cell-mediated immunity, thereby offering broader protection compared with single-component vaccines [1, 23, 26]. The dual-component formulation (bacterin plus toxoid) resulted in higher antibody titers, no mortalities, and no CLA abscesses compared with single-component vaccines, demonstrating superior protective efficacy in our study. However, the group administered bacterin alone or the group administered insufficient toxoid dose had relatively low antibody titers and survival rates, and the surviving individuals after challenge vaccination had high levels of antibodies to PLD, suggesting that the immune response targeting PLD, a toxin important in the pathogenesis and spread of CLA, is key to the vaccine’s protective effect (Figure 3D).

In addition, this study is significant in that it developed a vaccine for goat infectious diseases and established a model for evaluating it. Previous CLA vaccine studies were mainly focused on sheep, so there was a relative lack of data on vaccine administration and infection model establishment in goats. In this study, we standardized the challenge dose (1 × 10^6^ CFU/mL) using strains derived from domestic goat breeding sites and monitored the occurrence of major lesions (pulmonary and cephalic lymph node abscesses) after infection, thereby establishing a highly reproducible model that can be used for future CLA vaccine studies and laboratory infection tests (Figure 3).

In addition, by conducting infection experiments in 3–4 month-old goats, we confirmed that the period of heightened susceptibility following the loss of maternal antibodies could be effectively targeted through vaccination. Several studies have reported that maternally derived antibodies in goats decline substantially by 8–10 weeks of age, with near-complete loss by 2.5 months, thereby reducing interference with vaccine-induced immune responses [27]. CLA is mainly evident in animals aged 6 months to 1 year or older, but considering that latent infection in the body can occur from an early age, it suggests that administering the vaccine at this time (3–4 months) is very effective in controlling CLA. Above all, in the field trial results of this study, no adverse reactions occurred after vaccination, and the vaccinated group maintained a high antibody titer stably in all farms. In particular, while clinical abscesses occurred in naturally infected animals (control group) on some farms (farm B), no similar cases were reported in the vaccinated group during the same period (Figure 5). This indicates that this vaccine is sufficiently safe and effective in the field environment. In addition, a similar level of antibody titer increase was observed in a farm raising sheep (farm C), showing that this vaccine could be used with causation in sheep as well (Figure 5).

Nevertheless, it should be recognized that *C. pseudotuberculosis* is not always the sole pathogen isolated from CLA-like lesions. Previous abattoir and field studies have reported variable detection rates of *C. pseudotuberculosis*, ranging from 12.6% to 48.3% of lymph nodes, while other bacteria such as *Staphylococcus aureus* subspecies *anaerobius* were found as predominant agents in some regions, including Spain (44.4%) [4, 12, 28–30]. This epidemiological variability suggests that the universality of CLA vaccines may be limited in areas where multiple pathogens contribute to lesion development. Nonetheless, given that *C. pseudotuberculosis* remains the principal agent in most endemic regions, vaccines targeting this bacterium are expected to significantly reduce disease burden in goats and sheep.

However, this study has some points that need to be supplemented. For example, as this study focused on 3–4 month-old animals, it can effectively prevent the period of acquiring infection during early life, but the vaccine effect on other animals, such as pregnant and lactating animals, and elderly animals remains to be studied in follow-up studies. In addition, large-scale follow-up investigations on long-term immunity persistence and cross-protection effects in situations of infection with various domestic and foreign mutant strains remain as future research tasks. In summary, this study developed a bacterin–toxoid combination vaccine using a Korean native goat strain and established farm and laboratory models, targeting it to secure evidence that it is effective in preventing CLA. This not only provides a new solution to CLA control, which is a major problem in the domestic goat industry, but also provides important implications for the development of a vaccine platform in goats. In the future, it is expected that the commercial usability of this vaccine will be further strengthened through large-scale field application and long-term immunological monitoring, and that this will also spur the development of other goat infectious disease vaccines.

## Supplementary Information

**Additional file 1** ***Establishment of a Corynebacterium pseudotuberculosis challenge model in goats. ***Investigation of survival and CLA abscess formation according to the dose of *Corynebacterium pseudotuberculosis* challenge.

**Additional file 2** ***Results of clinical symptoms and adverse reactions to vaccines for the field safety evaluation of vaccine prototypes.***

## Data Availability

No datasets were generated or analyzed during the current study.

## Abbreviations

AP: alkaline phosphatase
APQA: Animal and Plant Quarantine Agency
BCG: Bacillus Calmette–Guérin
BHI-T: Brain Heart Infusion broth supplemented with 0.05% Tween 80
CLA: caprine caseous lymphadenitis
CFU: colony-forming unit
HRP: horseradish peroxidase
IACUC: Institutional Animal Care and Use Committee
NBT/BCIP: a nitro blue tetrazolium/5-bromo-4-chloro-3-indolyl phosphate
PBS: phosphate-buffered saline
PBS-T: phosphate-buffered saline containing 0.1% Tween 20
PLD: phospholipase D
SD: standard deviation
SDS–PAGE: sodium dodecyl–sulfate polyacrylamide gel electrophoresis
SPF: specific-pathogen-free
TMB: 3,3′,5,5′-tetramethylbenzidine
