# Supplementary material for: Development of a bacterin–toxoid vaccine using a Korean isolate for protection against caseous lymphadenitis in goats
Source: Vet Res. 2026 Jan 3;57:22. doi: 10.1186/s13567-025-01685-8 (PMC12857065; doi:10.1186/s13567-025-01685-8)
Supplement: Supplementary file 1 — Additional file 1 Establishment of a Corynebacterium pseudotuberculosis challenge model in goats. Investigation of survival and CLA abscess formation according to the dose of Corynebacterium pseudotuberculosis challenge. [file 13567_2025_1685_MOESM1_ESM.docx]

**Supplementary Information**

| **Group** | **Titer**  **(CFU/mL)** | **No. of**  **goats** | **No. of**  **total**  **death** | **Death (No. of goats)** | | | | **CLA abscesses (No. of goats)** | | | | |
| --- | --- | --- | --- | --- | --- | --- | --- | --- | --- | --- | --- | --- |
|  |  |  |  | **1w** | **2w** | **3w** | **4w** | **Lung** | **Lymph**  **nodes** | **Spleen** | **Kidney** | **Liver** |
| **1** | **1x10^8.0^** | 4 | 4 | 4 | - | - | - | 0 | 0 | 0 | 0 | 0 |
| **2** | **1x10^7.0^** | 4 | 4 | 3 | 1 | - | - | 1 | 0 | 0 | 0 | 0 |
| **3** | **1x10^6.0^** | 4 | 3 | 0 | 2 | 1 | 0 | 2 | 3 | 0 | 0 | 0 |
| **4** | **1x10^5.0^** | 4 | 1 | 0 | 1 | 0 | 0 | 2 | 1 | 0 | 0 | 0 |
| **5** | **1x10^4.0^** | 4 | 1 | 0 | 0 | 0 | 1 | 1 | 3 | 0 | 0 | 0 |
| **6** | **Control** | 4 | 0 | 0 | 0 | 0 | 0 | 0 | 0 | 0 | 0 | 0 |

**Table 1. Establishment of a *Corynebacterium pseudotuberculosis* challenge model in goats.** Investigation of survival and CLA abscess formation according to the dose of *Corynebacterium pseudotuberculosis* challenge.
