# Supplementary material for: Development of a bacterin–toxoid vaccine using a Korean isolate for protection against caseous lymphadenitis in goats
Source: Vet Res. 2026 Jan 3;57:22. doi: 10.1186/s13567-025-01685-8 (PMC12857065; doi:10.1186/s13567-025-01685-8)
Supplement: Supplementary file 2 — Additional file 2 Results of clinical symptoms and adverse reactions to vaccines for the field safety evaluation of vaccine prototypes. [file 13567_2025_1685_MOESM2_ESM.docx]

**Supplementary Information**

**Table 2. Results of clinical symptoms and adverse reactions to vaccines for the field safety evaluation of vaccine prototypes**

| **Farm** | **Vaccination** | **Group** | **Clinical signs** | | | |
| --- | --- | --- | --- | --- | --- | --- |
|  |  |  | **Changes in activity/mobility** | **Suppuration** | **Necrosis** | **Death** |
| **A** | **1^st^ vaccination** | **Vaccination** | **-** | **-** | **-** | **-** |
|  |  | **Non-vaccination** | **-** | **-** | **-** | **-** |
|  | **2^nd^ vaccination** | **Vaccination** | **-** | **-** | **-** | **-** |
|  |  | **Non-vaccination** | **-** | **-** | **-** | **-** |
| **B** | **1^st^ vaccination** | **Vaccination** | **-** | **-** | **-** | **-** |
|  |  | **Non-vaccination** | **-** | **+^*^** | **-** | **-** |
|  | **2^nd^ vaccination** | **Vaccination** | **-** | **-** | **-** | **-** |
|  |  | **Non-vaccination** | **-** | **+^*^** | **-** | **-** |
| **C** | **1^st^ vaccination** | **Vaccination** | **-** | **-** | **-** | **-** |
|  |  | **Non-vaccination** | **-** | **-** | **-** | **-** |
|  | **2^nd^ vaccination** | **Vaccination** | **-** | **-** | **-** | **-** |
|  |  | **Non-vaccination** | **-** | **-** | **-** | **-** |

^*^+, Caseous lymphadenitis-like lesions were confirmed in two of five goats, and abscesses occurred 31 and 45 days after vaccination, respectively.
